# Botulinum Toxin (Dysport) to Prevent Radiation‐Induced Dysfunction of Salivary Glands in Head and Neck Cancer

**DOI:** 10.1002/cnr2.70576

**Published:** 2026-05-19

**Authors:** Farshid Farhan, Iraj Eyvaz‐zadeh, Shayan Forghani, Amirhossein Shahsavand, Reza Samiee, Sepideh Mansouri, Mehrshad Abbasi, Ali Kazemian, Mahdi Aghili, Mehrdad Jafari, Ebrahim Karimi, Ebrahim Esmati, Fatemeh Jafari

**Affiliations:** ^1^ Radiation Oncology Department, Cancer Institute, Imam‐Khomeini Hospital Complex (IKHC) Tehran University of Medical Sciences Tehran Iran; ^2^ Radiation Oncology Research Center (RORC), Imam Khomeini Hospital Complex Tehran Iran; ^3^ Department of Nuclear Medicine, Vali‐Asr Hospital Tehran University of Medical Sciences Tehran Iran; ^4^ Otolaryngology Research Center, Imam Khomeini Hospital Tehran University of Medical Sciences Tehran Iran; ^5^ Department of Otolaryngology, Amir‐Alam Hospital Tehran University of Medical Sciences Tehran Iran; ^6^ Radiology Department Tehran University of Medical Sciences Tehran Iran

**Keywords:** botulinum toxins, head and neck neoplasms, radiotherapy, xerostomia

## Abstract

**Background:**

The main goal of this pilot trial was to investigate the efficacy of botulinum toxin (Dysport) injection before radiotherapy in preserving salivary gland secretion in patients with head and neck cancer.

**Methods:**

Sixteen patients with head and neck cancer were enrolled, 9 received 50 IU Dysport, and 7 recieved NaCl (0.9%) into the submandibular glands before chemo‐radiotherapy. To investigate the salivary gland function, technetium pertechnetate salivary gland scintigraphy, as well as saliva accumulation and the University of Washington Quality of Life (UW‐QoL) Questionnaire were utilized “before” and “6 months after the end” of radiotherapy.

**Results:**

Sixteen patients were randomized (9 botulinum toxin, 7 placebo). Baseline characteristics were comparable between groups. At 6 months post‐radiotherapy, basal saliva volume was higher in the botulinum toxin group (median 5.0 cc vs. 2.0 cc; *p* < 0.001) as was stimulated saliva volume (6.0 cc vs. 2.0 cc; *p* < 0.001). UW‐QoL scores were significantly lower, indicating better quality of life, in the botulinum toxin group (37.0 vs. 67.0; *p* < 0.001). Covariate‐adjusted analyses confirmed botulinum toxin as the only significant predictor of saliva preservation and improved QoL.

**Conclusion:**

A single injection of botulinum toxin before radiotherapy preserves salivary gland function and improves quality of life. Based on these findings, we advocate for a multicenter Phase II trial to define the optimal dose–response relationship and determine the ideal timing for injection relative to the start of radiotherapy.

## 1 Introduction

Radiotherapy, either as monotherapy or in combination with other modalities, is a well‐established curative treatment for head and neck cancers (HNC) [1, 2, 3]. Because of the close location of salivary glands to tumor sites, they are often exposed to radiation, which can lead to several complications [4, 5, 6]. Among these, xerostomia is one of the most common and distressing side effects, increasing the risk of oral and dental problems and reducing patients' quality of life [5, 7].

Despite many experimental and clinical studies, no agent with reliable protective effects against radiation‐induced salivary gland damage has been identified [5, 6]. Botulinum toxin (BTX), produced by *Clostridium botulinum* , is a well‐studied biological agent with a wide safety margin. It has established applications in neurological diseases such as myasthenia gravis [8], and in salivary gland disorders such as sialorrhea [9]. Injection of BTX into the parotid and submandibular glands has been shown to reduce saliva production by decreasing muscarinic receptor expression and inducing temporary apoptosis in glandular cells, with function usually recovering after about 12 weeks [10, 11, 12].

In addition to controlling sialorrhea, BTX has been studied for reducing radiation‐induced fibrosis, preventing muscle spasm, and lowering the risk of salivary fistula after oncologic surgery [13, 14]. Preclinical studies in rats demonstrated that intraglandular BTX could significantly reduce radiation‐induced salivary gland toxicity [15, 16]. A subsequent phase I clinical trial in HNC patients confirmed these protective effects in humans [17]. According to the Law of Bergonié and Tribondeau, cells with higher mitotic activity are more sensitive to radiation. By inducing cell cycle arrest in the G0–G1 phase, BTX may increase radio resistance [18]. Following previous preclinical and phase I clinical studies, we evaluated herein the efficacy of pre‐radiation injection of BTX in submandibular salivary glands to preserve glandular function of HNC patients receiving radiation therapy in our center. Based on these findings, we aimed to evaluate whether pre‐radiation injection of BTX into the submandibular glands could help preserve salivary function in HNC patients receiving radiotherapy in our center.

## 2 Method

### 2.1 Study Design

This was a pilot study, performed in patients referred to our center. The main objective of the study was to assess the efficacy of BTX in preserving the function of salivary glands in patients with HNC following radiotherapy, since we will have more cells of the submandibular gland in the G0–G1 phase with botulinum injection. This study has been registered in the Iranian Registry of Clinical Trials (code: IRCT20180225038862N1). The study was approved by the Research Council and the Ethical Committee and performed entirely in accordance with the guidelines of good clinical practice and the Declaration of Helsinki. Written informed consents were acquired from all patients enrolled before study performance.

### 2.2 Patient Selection and Sample Size Calculation

Patients with histopathologically confirmed HNC were considered eligible for the study if the baseline function of the salivary glands was normal and symmetric. Before enrollment, MRI scans were reviewed to rule out tumor involvement of the submandibular and parotid glands. Furthermore, patients had to be planned to receive a total of 60–70 Gy of radiation to both submandibular glands within the radiation field, with a minimum dose of 40 Gy. Patients must be at least 18 years of age at the time of the study. Exclusion criteria of the study consisted of submandibular gland extirpation, any previous history of radiotherapy, alcohol consumption, opium addiction, abnormalities associated with salivary glands, pregnancy, and consumption of anticholinergic agents or medications potentially affecting the secretory function of glands.

Since the sample size for pilot studies is a matter of controversy, and no exact formula exists, we decided to include the patients available at the time, meeting the conditions above, in our study.

### 2.3 Randomization and Treatment With BTX

A total of 19 patients were enrolled in the study, and were divided into control (*n* = 9) and treatment group (*n* = 10) through block randomization. Before intervention, the baseline function of the salivary glands was recorded by scintigraphy. Furthermore, patients were requested to accumulate basal and stimulated saliva secretion after using 2 cc of lemon juice. In addition, the xerostomia‐associated quality of life (XeQoLs) of patients was assessed through the University of Washington Quality of Life Questionnaire (UW‐QoL). Patients in the treatment group received 50 IU BTX (Dysport) by direct injection into the submandibular salivary glands. Patients in the control group received NaCl (0.9%) with the same volume and through the same procedure as in the treatment group. Injections were performed under ultrasound guidance 2–3 weeks before initiating radiotherapy, based on results observed with the administration of BTX in previous studies [17]. Block random allocation was performed using Excel software before initiation of the study, and results were saved in sealed opaque envelopes.

This study was conducted in a double‐blind design. Both the patients and the outcome assessors were blinded to treatment allocation. The investigators responsible for saliva collection, scintigraphic evaluation, and scoring of the UW‐QoL questionnaire were not aware of the group assignments. To preserve blinding, injections were administered using identically prepared syringes containing either botulinum toxin or normal saline, prepared and coded by a hospital pharmacist. Data analysis was not performed in a blinded manner. Six months following chemoradiotherapy, patients were again subjected to quantitative scintigraphic studies, accumulation of saliva, and assessment of XeQoLs through filling out the same questionnaire.

### 2.4 Endpoints and Outcomes Measurements

#### 2.4.1 Saliva Accumulation

First, patients were asked to empty their basal saliva into a graduated tube within five minutes. In the same way, excited saliva was collected five minutes after swallowing lemon juice.

#### 2.4.2 University of Washington Quality of Life Questionnaire

One of the primary outcome measures of the present study was the change in scores between the UW‐QoL Questionnaire before and 6 months after chemoradiotherapy. The UW‐QoL is a validated instrument specifically designed for HNC patients and evaluates multiple domains related to health‐related quality of life [19]. The UW‐QoL is a validated instrument specifically developed for patients with head and neck cancer and evaluates several domains related to health‐related quality of life, including pain, appearance, activity, recreation, swallowing, chewing, speech, shoulder function, taste, saliva, mood, and anxiety.

Each domain includes five ordered response options reflecting increasing levels of dysfunction. The responses are converted to a standardized 0–100 scale, according to the UW‐QoL scoring guidelines. On this scale, 0 represents the best possible condition (no impairment) and 100 represents the worst possible condition (maximum impairment). Therefore, higher scores indicate poorer quality of life, whereas lower scores indicate better functional status and fewer symptoms.

For the purposes of the present study, particular attention was given to the saliva‐related domains assessing the amount and consistency of saliva, as these parameters are directly associated with xerostomia following chemoradiotherapy. Changes in UW‐QoL scores between baseline and the 6‐month follow‐up were used to evaluate the impact of the intervention on xerostomia‐related quality of life.

#### 2.4.3 Quantitative Scintigraphic Studies

Briefly, after confirmation that patients were well hydrated, a bolus of 259 MBq (7 mCi) 99mTc‐sodium pertechnetate was injected intravenously. A mobile scintillation camera (Triad XLT; Trionox Research Laboratory, Twinsburg, Ohio) with a small field of view was used to obtain images up to 40 min after injection (128 × 128–pixel matrix at 60 s per frame). To measure excretory function, oral stimuli (2 mL of lemon juice) were administered intraorally, while patients were asked to refrain from any mechanical stimulation (swallowing or chewing) of salivary flow during the imaging. Regions of interest were marked over the parotid and submandibular glands, and corresponding time‐activity curves were drawn to calculate the functional parameters: maximal accumulation (%), ejection fraction (%), and the pre‐stimulatory and post‐stimulatory oral index (%). Maximum accumulation was defined as the value of (peak activity before stimulation—initial activity at 1 min after radiotracer injection) divided by peak activity before stimulation. The response of the salivary gland to lemon juice was noted on the time activity curve as a sharp decline in the activity in the gland with a subsequent slow buildup. Then, the ejection fraction in each salivary gland was calculated as the amount of radioactivity cleared from the gland divided by the preclearance activity. The semiquantitative oral radioactivity index was also calculated from the time‐activity curve.

### 2.5 Statistical Analysis

An intent‐to‐treat analysis was performed on all randomized patients. Statistical analyses were conducted using R software. Continuous variables were summarized as median and interquartile range (IQR), while categorical variables were reported as counts and percentages. Baseline characteristics between the treatment and placebo groups were compared using the Wilcoxon rank‐sum test for continuous variables and the chi‐squared or Fisher's exact test for categorical variables.

The primary analysis of treatment efficacy was conducted using a non‐parametric analysis of covariance (ANCOVA). This was performed by applying a linear model to the ranks of the post‐treatment outcome variables (basal saliva, stimulated saliva, and quality of life scores). The models adjusted for the rank of the corresponding baseline measurement and the mean radiation dose to the submandibular gland. The effect size for the treatment was quantified using the rank‐biserial correlation, which measures the difference between the proportion of favorable and unfavorable pairings in the treatment and placebo groups. A *p* value of less than 0.05 was considered statistically significant for all analyses.

## 3 Results

### 3.1 Baseline Characteristics

**FIGURE 1:**
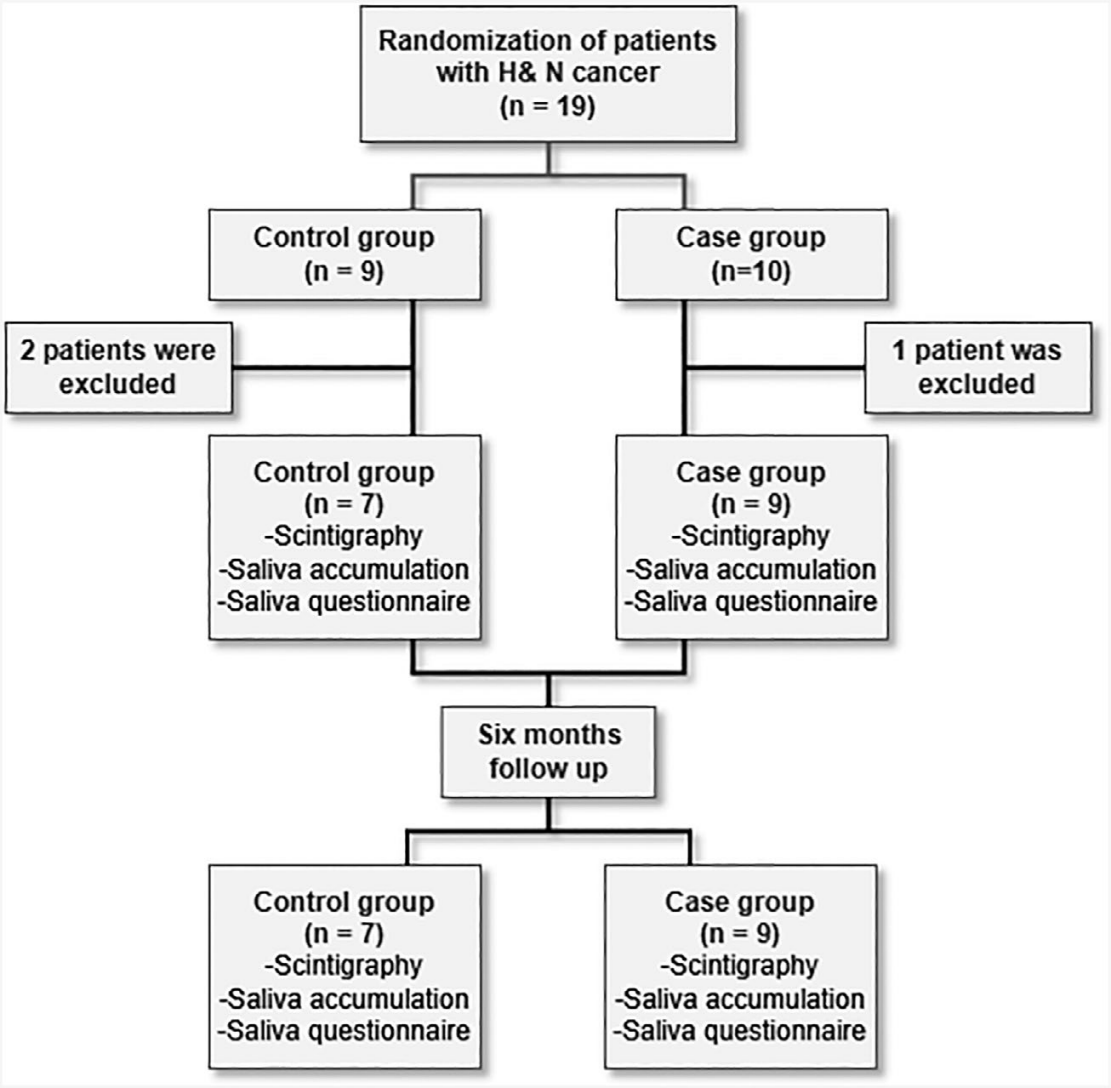
CONSORT flow diagram of patient enrollment, allocation, and analysis.

A total of 16 patients were included in the final analysis, with 9 in the botulinum toxin group and 7 in the placebo group. Patient enrollment and allocation are summarized in the CONSORT diagram (Figure 1). The baseline demographic and clinical characteristics are shown in Table 1. The two groups were well matched in terms of age, with a median of 50 years in both. Most patients in both groups were male. The distributions of tumor size (AJCC), lymph node status, and primary tumor location—most commonly the oral cavity—were similar. There were no significant differences in prescribed CTV dose or mean dose delivered to the submandibular gland. The proportion of patients receiving chemotherapy was also comparable (56% in the botulinum toxin group vs. 43% in the placebo group; *p* > 0.9). In addition, no statistically significant differences were observed at baseline in basal or stimulated saliva secretion, or in UW‐QoL scores.

**TABLE 1:** Baseline patient and treatment characteristics by study group.

Characteristic	Placebo *N* = 7^a^	Botulinum toxin *N* = 9^a^	*p* ^b^
Age (years)	50.0 (42.0–63.0)	50.0 (49.0–62.0)	0.7
Gender (female)	2 (29%)	1 (11%)	0.6
Tumor size (AJCC)			0.8
2	3 (43%)	4 (44%)	
3	1 (14%)	3 (33%)	
4	3 (43%)	2 (22%)	
Lymph node status (AJCC)			0.5
0	4 (57%)	3 (33%)	
1	1 (14%)	4 (44%)	
2	2 (29%)	1 (11%)	
3	0 (0%)	1 (11%)	
Tumor location			0.8
Oral	3 (43%)	4 (44%)	
nasopharyngeal	2 (29%)	1 (11%)	
Laryngeal	2 (29%)	4 (44%)	
Mean CTV dose (Gy)			0.6
60	3 (43%)	2 (22%)	
66	0 (0%)	2 (22%)	
70	4 (57%)	5 (56%)	
Mean submandibular dose (Gy)	44.1 (43.7–45.3)	43.0 (42.2–45.2)	0.4
Received chemotherapy	3 (43%)	5 (56%)	> 0.9
Baseline basal saliva (cc)	2.0 (2.0–4.0)	3.0 (1.0–4.0)	> 0.9
1	0 (0%)	3 (33%)	0.2
2	4 (57%)	1 (11%)	
2.5	1 (14%)	1 (11%)	
3	2 (29%)	4 (44%)	
Baseline stimulated saliva (cc)	4.0 (2.0–4.0)	4.0 (3.0–5.0)	0.4
5	1 (14%)	1 (11%)	0.9
6	2 (29%)	1 (11%)	
6.5	0 (0%)	1 (11%)	
7	3 (43%)	3 (33%)	
7.5	1 (14%)	1 (11%)	
8	0 (0%)	2 (22%)	
Baseline UW‐QoL score	2.0 (1.0–3.0)	3.0 (2.0–3.0)	0.2
18	2 (29%)	1 (11%)	0.7
19	2 (29%)	2 (22%)	
20	3 (43%)	4 (44%)	
21	0 (0%)	2 (22%)	

^a^ Median (Q1—Q3); *n* (%).

^b^ Wilcoxon rank sum test; Fisher's exact test.

### 3.2 Primary Outcomes at 6 Months Post‐Radiotherapy

The primary outcomes measured 6 months after radiotherapy are presented in Table 2.

**TABLE 2:** Post‐radiotherapy saliva output and quality of life outcomes by study group.

Characteristic	Placebo^a^ *N* = 7	Botulinum toxin^a^ *N* = 9	*p* ^b^
Basal saliva after RT (cc)	2.0 (2.0–2.0)	5.0 (4.0–7.0)	< 0.001
0	1 (14%)	0 (0%)	< 0.001
0.1	6 (86%)	0 (0%)	
0.3	0 (0%)	1 (11%)	
0.4	0 (0%)	3 (33%)	
0.5	0 (0%)	1 (11%)	
0.7	0 (0%)	1 (11%)	
0.8	0 (0%)	3 (33%)	
Stimulated saliva after RT (cc)	2.0 (1.0–2.0)	6.0 (5.0–7.0)	< 0.001
0.2	2 (29%)	0 (0%)	0.001
0.3	4 (57%)	0 (0%)	
0.4	1 (14%)	0 (0%)	
1	0 (0%)	2 (22%)	
1.2	0 (0%)	1 (11%)	
1.3	0 (0%)	3 (33%)	
1.5	0 (0%)	3 (33%)	
UW‐QoL score after RT	67.0 (56.0–71.0)	37.0 (35.0–39.0)	< 0.001

^a^ Median (Q1—Q3); *n* (%).

^b^ Wilcoxon rank sum test; Fisher's exact test.

#### 3.2.1 Saliva Secretion

**FIGURE 2:**
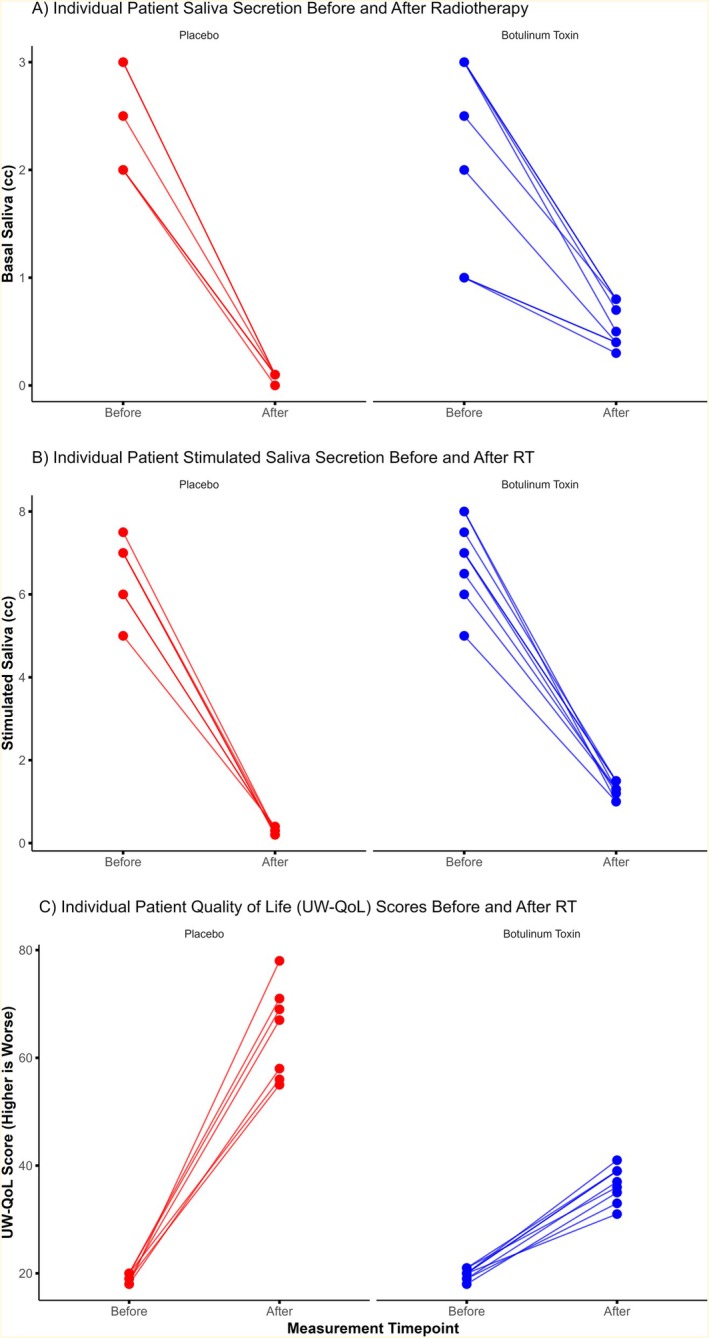
Individual patient changes in (A) basal saliva secretion, (B) stimulated saliva secretion, and (C) University of Washington Quality of Life (UW‐QoL) scores from baseline to 6 months after radiotherapy.

**FIGURE 3:**
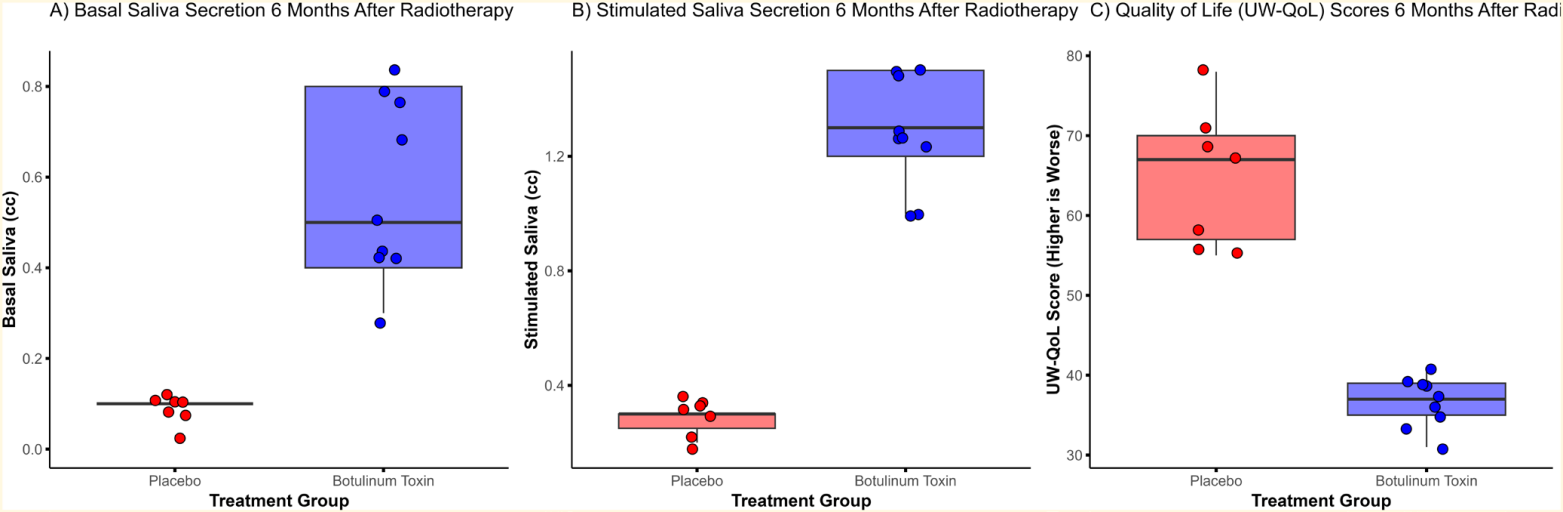
Comparison of (A) basal saliva secretion, (B) stimulated saliva secretion, and (C) University of Washington Quality of Life (UW‐QoL) scores between the Botulinum Toxin and Placebo groups at 6 months after radiotherapy. (Higher UW‐QoL scores indicate worse quality of life.) Data are presented as box plots.

Significant differences were found between the two groups in both basal and stimulated saliva volumes. The median basal saliva volume at 6 months was 5.0 cc (IQR 4.0–7.0) in the botulinum toxin group compared to 2.0 cc (IQR 2.0–2.0) in the placebo group (*p* < 0.001). For stimulated saliva, the median value was 6.0 cc (IQR 5.0–7.0) in the botulinum toxin group versus 2.0 cc (IQR 1.0–2.0) in the placebo group (*p* < 0.001). The individual changes from baseline to post‐radiotherapy are shown in Figure 2, and the distributions of post‐radiotherapy saliva volumes are presented in Figure 3.

#### 3.2.2 Quality of Life (UW‐QoL)

A statistically significant difference was also observed in UW‐QoL scores at 6 months. The median score was 37.0 (IQR 35.0–39.0) in the botulinum toxin group and 67.0 (IQR 56.0–71.0) in the placebo group (*p* < 0.001). Changes in UW‐QoL scores for individual patients are presented in Figure 2, and group distributions are displayed in Figure 3.

### 3.3 Covariate‐Adjusted Analysis of Primary Outcomes

A non‐parametric ANCOVA on ranked outcome data was performed, adjusting for baseline values and mean radiation dose to the submandibular gland. For basal saliva secretion, botulinum toxin remained the most statistically significant predictor of higher ranks (*p* < 0.001). Baseline saliva secretion was also significant (*p* = 0.003), while the mean submandibular dose was not (*p* = 0.76). For stimulated saliva secretion, botulinum toxin was the only significant predictor (*p* < 0.001). Neither baseline secretion (*p* = 0.84) nor gland dose (*p* = 0.91) was significant. For UW‐QoL, botulinum toxin remained strongly associated with lower scores (*p* < 0.001). A non‐significant trend was observed for higher radiation dose and worse QoL (*p* = 0.079).

### 3.4 Tumor‐Site Stratified Analysis

To determine whether tumor location influenced treatment effects, patients were stratified by primary tumor site (oral cavity vs. nasopharyngeal/laryngeal). Across both subgroups, patients treated with botulinum toxin demonstrated higher basal and stimulated salivary flow and lower UW‐QoL scores at 6 months compared with the placebo group (Supplementary Figures S1 and S2).

To evaluate potential confounding from tumor site heterogeneity, a sensitivity analysis was performed in which tumor location was included as an additional covariate in the rank‐based ANCOVA models, adjusting for baseline values and mean radiation dose to the submandibular glands. Botulinum toxin remained a significant independent predictor of basal saliva (*p* < 0.001), stimulated saliva (*p* < 0.001), and UW‐QoL scores (*p* < 0.001). Tumor site was not associated with any outcome (not significant).

## 4 Discussion

This pilot randomized controlled trial provides preliminary evidence that a single, pre‐radiotherapy botulinum toxin (Dysport) injection into the submandibular glands may significantly preserve salivary function and improve quality of life in patients with head and neck cancer six months post‐treatment. These findings point to a potential way of mitigating salivary dysfunction and subsequent xerostomia, which affects up to 80% of patients and severely impacts their quality of life, even with modern radiotherapy techniques [20].

The mechanism underlying this radioprotective effect is likely multifactorial, combining established radiobiological principles with recent immunological insights [16]. The primary action of botulinum toxin is a temporary chemodenervation that blocks presynaptic acetylcholine release, thereby inhibiting glandular secretion and inducing a state of functional quiescence [15]. By inducing this quiescent state, botulinum toxin may force acinar cells into the more radioresistant G0/G1 phases of the cell cycle [15]. Furthermore, preclinical work suggests botulinum toxin also exerts an anti‐inflammatory effect by reducing post‐irradiation neutrophil infiltration and attenuating the pro‐inflammatory cytokine response, specifically downregulating CXCL5 [16]. This reduction in acute inflammation may prevent the cascade of events leading to chronic fibrosis and permanent glandular damage. This functional shutdown is also associated with a profound decrease in glandular metabolic activity, which may further contribute to radioresistance [21]. Importantly, the evidence regarding the role of these pathways and their associations with our findings remains hypothetical and requires validation in studies with proper design.

Our findings represent a clinical translation of preclinical data, which demonstrated that prophylactic botulinum toxin preserved glandular histology and function in irradiated rodent models [15, 16]. While a prior Phase I human trial established the safety of botulinum toxin injection, it did not demonstrate significant efficacy [17]. This may be attributable to its primary endpoint of salivary gland scintigraphy, an endpoint that measures radionuclide uptake but may not correlate well with the clinically crucial function of saliva production [22, 23, 24]. Our use of direct saliva volume measurement and the validated UW‐QoL outcome allowed us to directly evaluate salivary dysfunction and its impact, detecting a significant benefit that the earlier trial could not establish.

Additionally, while our study employed a non‐parametric ANCOVA to adjust for confounders and baseline values, Teymoortash et al. used a Simple Analysis of Change Scores to analyze their scintigraphy data [17]. The ANCOVA can often provide a more precise estimate of treatment effect, especially with smaller sample sizes, with significant correlation between variables [25, 26]. The difference in analytical methods could account for some of the inconsistencies between the outcomes of these studies.

This biological radioprotection strategy offers advantages over existing approaches. The primary systemic cytoprotectant, amifostine, has shown efficacy in reducing xerostomia but is limited by significant toxicities, including nausea, emesis, and hypotension [27]. Additionally, its protective effect is diminished in patients receiving concurrent chemoradiotherapy, which is the standard of care in many head and neck cancer settings and constitutes half of our study population [27]. While advanced techniques like intensity‐modulated radiation therapy and proton therapy have improved gland sparing, the decisive determinant of salivary function remains the absolute dose delivered to the gland [28]. Our dosimetric assessment was therefore centered on the submandibular glands, given their anatomical inclusion in the high‐dose target volume and their physiological role in baseline saliva production. We acknowledge that detailed parotid gland dosimetry (e.g., mean parotid dose or dose–volume constraints) was not consistently available, which limits our ability to fully characterize the parotid‐specific dose–response relationship for xerostomia in this cohort. Due to the anatomical proximity of the submandibular glands to critical lymph node targets, significant radiation doses are often unavoidable [29]. Botulinum toxin thus offers a complementary biological mechanism to protect glandular tissue that cannot be physically spared by advanced radiation planning. Other novel approaches, such as stem cell transplantation or gene therapy, despite demonstrated potential, remain experimental and are not yet available for widespread clinical application [2, 30].

Small sample size (*n* = 16) significantly limits the robustness and generalizability of our findings and our results regarding the efficacy and safety of botulinum toxin should be interpreted as explorative rather than confirmatory. Specifically, while our findings do not show a site‐specific pattern for the effects of botulinum toxin, our sample size may be underpowered to determine subtle effects. In addition, the single‐center design further limits generalizability, and the six‐month follow‐up period is insufficient to assess the long‐term durability of the protective effect, especially regarding late effects of radiotherapy on salivary glands.

However, the prospective, randomized, and placebo‐controlled design of this study provides a high level of evidence for a pilot investigation. A key strength is the use of clinically resonant endpoints: the objective measurement of salivary volume quantifies glandular function, while the UW‐QoL scale captures the patient's subjective experience of their health status. The observed 30‐point difference in median UW‐QoL scores is particularly important, as this magnitude of change represents a clinically meaningful improvement in patient well‐being [31].

## 5 Conclusion

This pilot randomized controlled trial provides early evidence that a single injection of botulinum toxin before radiotherapy may preserve salivary gland function and improve quality of life. Our findings provide context for a multicenter Phase II trial with an adequate sample size to investigate the optimal dose–response relationship and determine the ideal timing for injection relative to the start of radiotherapy. A longer follow‐up of at least 24 months is essential to establish the durability of the effect and monitor for any unforeseen long‐term adverse events.

## Author Contributions

**Farshid Farhan:** conceptualization, methodology, investigation, writing – original draft. **Shayan Forghani:** investigation, formal analysis, writing – original draft. **Iraj Eyvaz‐zadeh:** investigation, writing – original draft. **Sepideh Mansouri:** data curation, investigation. **Amirhossein Shahsavand:** investigation, writing – original draft, formal analysis. **Reza Samiee:** writing – original draft, investigation. **Ali Kazemian:** conceptualization, methodology. **Mahdi Aghili:** conceptualization, methodology. **Ebrahim Esmati:** investigation, writing – review and editing. **Fatemeh Jafari:** conceptualization, writing – review and editing, supervision, project administration. **Mehrdad Jafari:** investigation. **Mehrshad Abbasi:** investigation, resources. **Ebrahim Karimi:** investigation.

## Funding

The authors have nothing to report.

## Ethics Statement

The study was conducted according to the guidelines of the Declaration of Helsinki and approved by the Institutional Review Board of the Tehran University of Medical Sciences, Tehran, Iran (IR.TUMS.IKHC.REC.1396.4848). The trial was registered with the Iranian Registry of Clinical Trials (IRCT20180225038862N1).

## Conflicts of Interest

The authors declare no conflicts of interest.

## Supporting information


**FIGURE S1:** Individual trajectories of basal saliva secretion, stimulated saliva secretion, and UW‐QoL scores from baseline to 6 months, stratified by tumor site (oral cavity vs. nasopharyngeal/laryngeal) and treatment group.

**FIGURE S2:** Distribution of basal saliva, stimulated saliva, and UW‐QoL scores at 6 months after radiotherapy, stratified by primary tumor site (oral cavity vs. nasopharyngeal/laryngeal) and treatment group (botulinum toxin vs. placebo).

## Data Availability

The data that support the findings of this study are available from the corresponding author upon reasonable request.
