# Supplementary figures and images for: Botulinum Toxin (Dysport) to Prevent Radiation‐Induced Dysfunction of Salivary Glands in Head and Neck Cancer
Source: Cancer Rep (Hoboken). 2026 May 19;9(5):e70576. doi: 10.1002/cnr2.70576 (PMC13184827; doi:10.1002/cnr2.70576)

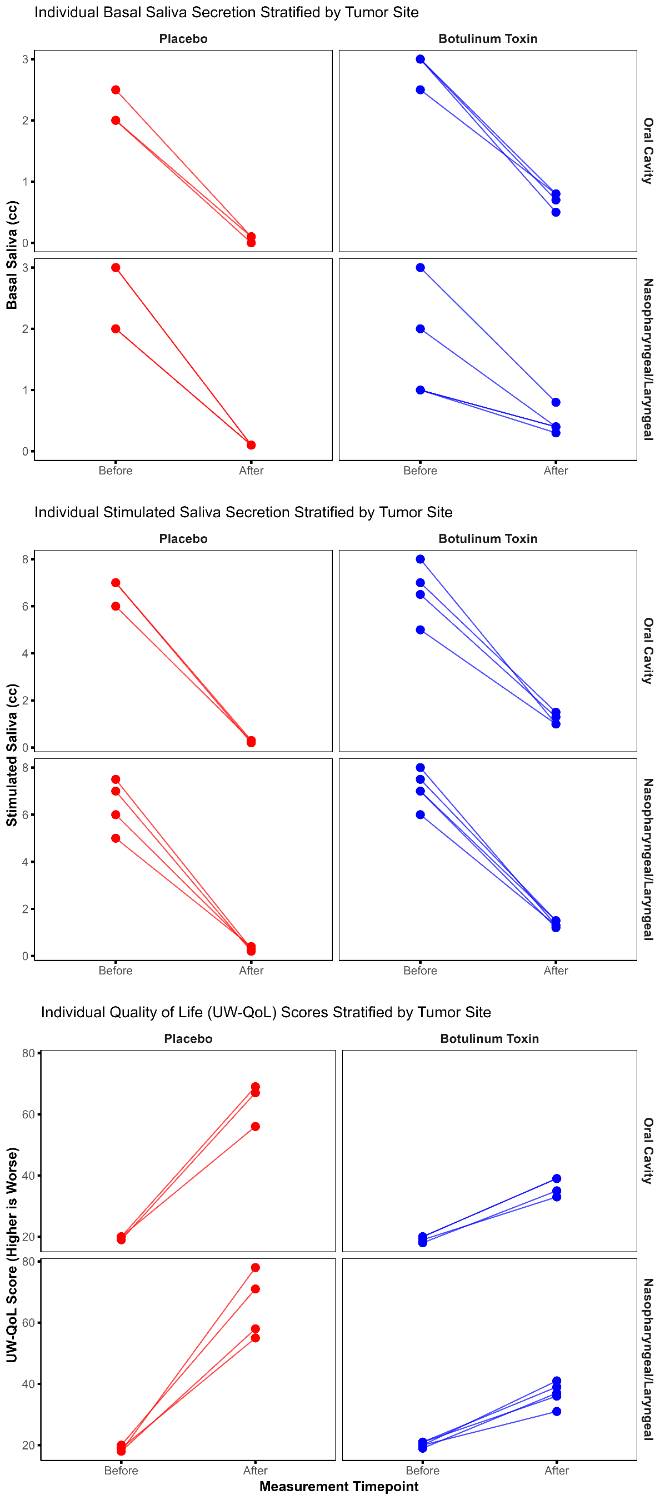

Supplement: Supplementary file 1 — FIGURE S1: Individual trajectories of basal saliva secretion, stimulated saliva secretion, and UW‐QoL scores from baseline to 6 months, stratified by tumor site (oral cavity vs. nasopharyngeal/laryngeal) and treatment group. [file CNR2-9-e70576-s002.jpeg]

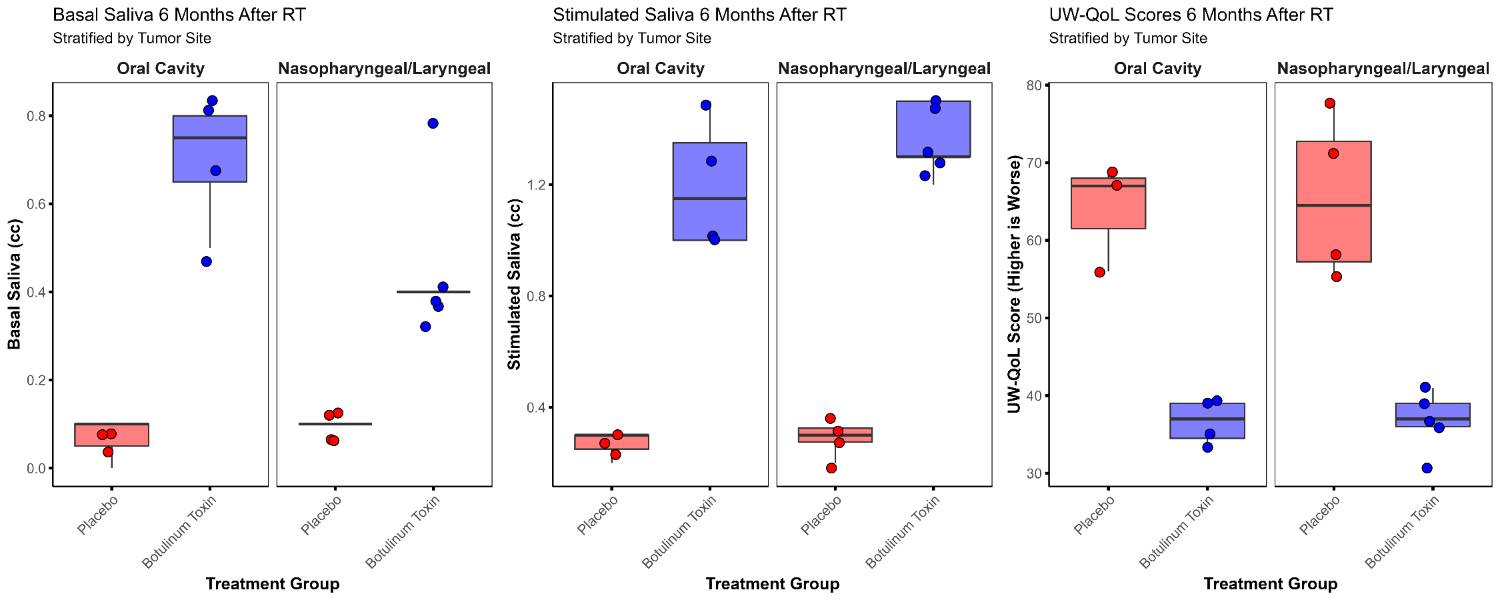

Supplement: Supplementary file 2 — FIGURE S2: Distribution of basal saliva, stimulated saliva, and UW‐QoL scores at 6 months after radiotherapy, stratified by primary tumor site (oral cavity vs. nasopharyngeal/laryngeal) and treatment group (botulinum toxin vs. placebo). [file CNR2-9-e70576-s001.jpeg]
